# Primary omental infarction – a benign cause of acute abdomen

**DOI:** 10.1515/pp-2023-0037

**Published:** 2024-05-13

**Authors:** Vikas Pemmada, Athish Shetty, Prakashini Koteshwar, Siddesh Rajpurohit, Ganesh Bhat

**Affiliations:** Department of Gastroenterology & Hepatology, 76793 Kasturba Medical College, Manipal Academy of Higher Education, Manipal, Karnataka, India; Department of Radiology, 76793 Manipal Academy of Higher Education, Manipal, Karnataka, India

**Keywords:** greater omentum, omental infarction, primary omental infarction, acute abdomen, radiology, omentectomy

## Abstract

**Objectives:**

Omental infarction (OI) is an uncommon cause of acute abdominal pain. A high index of clinical suspicion is required for diagnosis of OI as the incidence is less than 1 %, presenting with abdominal pain. We report primary OI’s clinical and radiological profile from a single tertiary care hospital in India.

**Methods:**

In this retrospective cross-sectional study, the electronic medical and radiology records of patients with abdominal pain were reviewed over seven years (2015–2022). Variables were systematically collected and analyzed.

**Results:**

A total of 22 patients diagnosed with primary OI were included in this study. Male preponderance (63.6 %) was noted with a mean age of 47.45 years (SD ± 13.84; range: 18–72 years). Most patients belonged to class I obesity (according to the Asia-Pacific body mass index classification) with a mean BMI of 26.56 kg/m^2^ (SD ± 3.21 kg/m^2^). All patients had abdominal pain as the primary symptom, with a mean duration of 8.64 days (SD ± 10.15; range: 1–42 days). The most common locations of pain were the right hypochondrium (27.3 %) and diffuse (27.3 %), followed by the right iliac fossa (18.1 %). Most (95.45 %, n=21/22) patients were treated conservatively, and only one required surgical intervention.

**Conclusions:**

Primary OI is a rare and benign cause of acute abdomen. Obesity is a risk factor but does not correlate with the size or severity of OI. Radiological imaging, like a computed tomography (CT) scan, is essential for diagnosis. A conservative management line should be the first approach in treating primary OI before considering surgical options.

## Introduction

The term omentum was thought to be derived from the Latin name “operimentum,” which means “covering.” The greater omentum is a large double-layered visceral peritoneal fold developed from the mesoderm [1, 2]. The omentum is rich in adipose tissue and has a surface area from 0.3 to 1.5 m^2^ covering the abdominal viscera. Morison called the omentum “abdominal policeman” in 1906 because of its ability to creep to areas of intra-abdominal insult. De Marchetti first reported acute torsion of the omentum in 1858. Historically, surgeons have noted intra-operative changes of the greater omentum in response to acute peritoneal inflammation or infection [2].

Omental infarction (OI) can be categorized as primary, spontaneous, or secondary based on the pathogenesis [3]. Often, there is no evident cause for primary or spontaneous OI. Secondary OI can occur due to systemic causes like hypercoagulable states, vasculitis, polycythemia, and torsion of the vascular pedicle due to local causes like intra-abdominal cysts, tumors, or adhesions. OI is a rare condition, with only a few hundred reported cases and fewer case series [4]. Radiological imaging using computed tomography (CT) and magnetic resonance imaging (MRI) has increased the yield of diagnosing OI. We report a retrospective case series of primary OI, analyzing the clinical and radiological profile.

## Materials and methods

All diagnosed cases of OI were included in this retrospective case series from 2015 to 2022 at a tertiary care referral center in India. Clinical and laboratory data was retrieved by reviewing the electronic medical case records. Radiological images were acquired from a Radiology Information & Picture Archiving and Communication System (RIS-PACS) software. Ethical approval was taken from the Institutional Ethics Committee [IEC approval number: IEC 1-397], and the study was registered at the Clinical Trials Registry of India (CTRI/2023/04/051624). Inclusion criteria included patients above 18 years of age and radiologically confirmed OI by a consultant radiologist. Patients with a secondary cause of OI were excluded from the study. Omental infarction was searched in the hospital RIS-PACS software using the keywords “omentum” and “infarction.” A total of 36 cases of OI were found over seven years. Fourteen were due to a secondary cause and were excluded. All the data of the 22 patients with primary OI were entered into Microsoft (MS) Excel and analyzed in SPSS V25. Patients were categorized as mild, moderate, and severe OI per their clinical course. Patients who were treated successfully on an OPD basis were considered as mild, requiring hospital admission as moderate and surgical intervention or intensive care unit (ICU) care as severe OI. Descriptive statistics were represented with percentages for qualitative data, mean with standard deviation (SD), or median with interquartile range (IQR) for quantitative data. The Shapiro-Wilk test was applied to find normality. Data included clinical presentations of patients, including duration and site of pain, laboratory parameters, radiological investigation(s), and management strategy. Non-parametric tests like the Mann-Whitney U test were used to compare means.

## Results

### Demographic data

Primary OI was more common in males (63.6 %) with a high male-to-female ratio of 1.75:1. A mean age of 47.45 years (SD ± 13.84; range: 18–72 years) was observed. 28.5 % (n=6) were overweight and 71.4 % (n=15) were obese. 61.9 % (n=13) belonged to class I, and 9.5 % (n=2) had class II obesity. The mean weight was 75.85 kg (SD ± 11.85; range: 60–101 kg) with a mean BMI of 26.56 kg/m^2^ (SD ± 3.21 kg/m^2^) (Table 1). One patient’s weight could not be retrieved and was not included in the above analysis.

**Table 1:** Baseline characteristics.

Variable	n (%)
Age	47.45 years (SD ± 13.84)
Gender	
Male	14 (63.6)
Female	08 (36.4)
Weight, kg	75.85 (SD ± 11.85)
BMI, kg/m^2^	26.56 (SD ± 3.21)
Symptoms	
Abdominal pain	22 (100)
Generalised/diffuse	06 (27.3)
Localised	14 (72.7)
Fever	02 (9)
Abdominal mass	01 (4.5)
Laboratory tests	
WBC, ×10^3^/μL	9.17 (SD ± 3.24)
ESR, mm/h	20.68

kg, kilograms; m, metres; mm, millimetres; h, hour; WBC, white blood cells; ESR, erythrocyte sedimentation rate.

### Clinical profile

**Figure 1:**
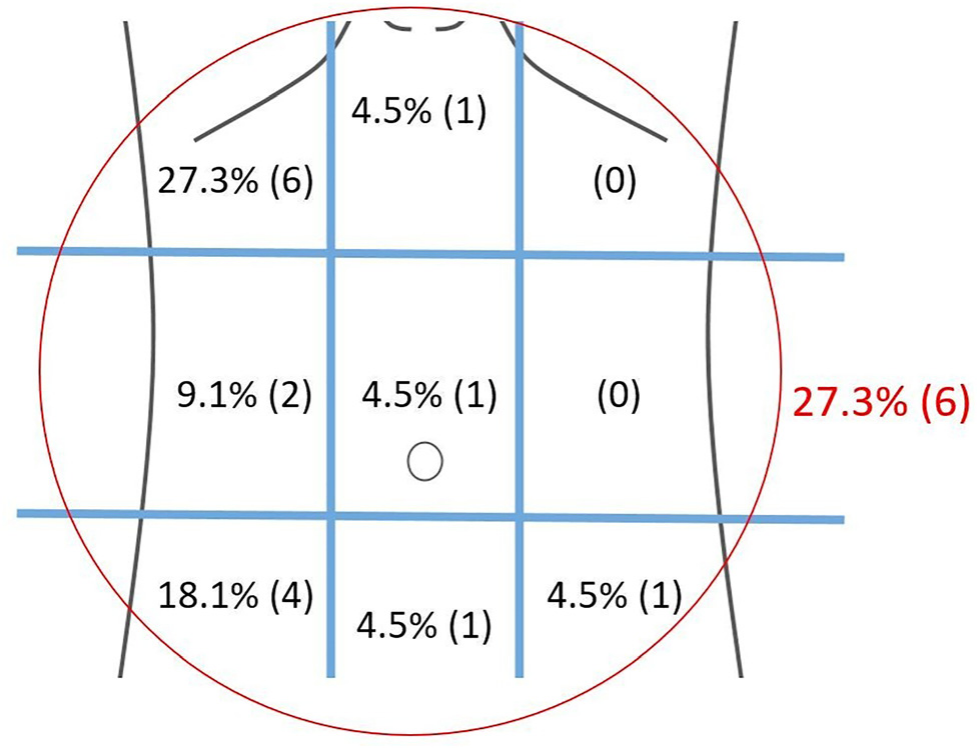
Distribution of pain in abdominal quadrants -localized pain in 72.7% and diffuse pain in 27.3% (in red).

Abdominal pain was the primary presentation in all (100 %) 22 patients. The mean duration of pain was 8.64 days (SD ± 10.15; range: 1–42 days). Compared to previous studies showing the right hypochondrium and right iliac fossa as the most typical pain sites, our study showed diffuse pain (27.3 %, n=6) and right hypochondrium (27.3 %, n=6) were the most common sites. They were followed by the right iliac fossa (18.1 %, n=4) and right lumbar (9.1 %, n=2). Uncommon sites included epigastric (4.5 %, n=1), hypogastric (4.5 %, n=1), umbilical (4.5 %, n=1), and left iliac fossa (4.5 %, n=1) (Figure 1). 9.09 % (n=2) presented with fever along with pain. One patient (4.5 %) presented with an abdominal mass in the epigastrium (Table 1).

### Investigations

#### Laboratory data

Raised white blood cell counts (WBC) and erythrocyte sedimentation rate (ESR) were seen among the blood investigations. The mean WBC was 9177 cells/μL (SD ± 3240; range: 4500–17,000 cells/μL). A mean ESR of 20.7 mm/h was observed with a maximum rate of 75.

#### Radiological data

**Figure 2:**
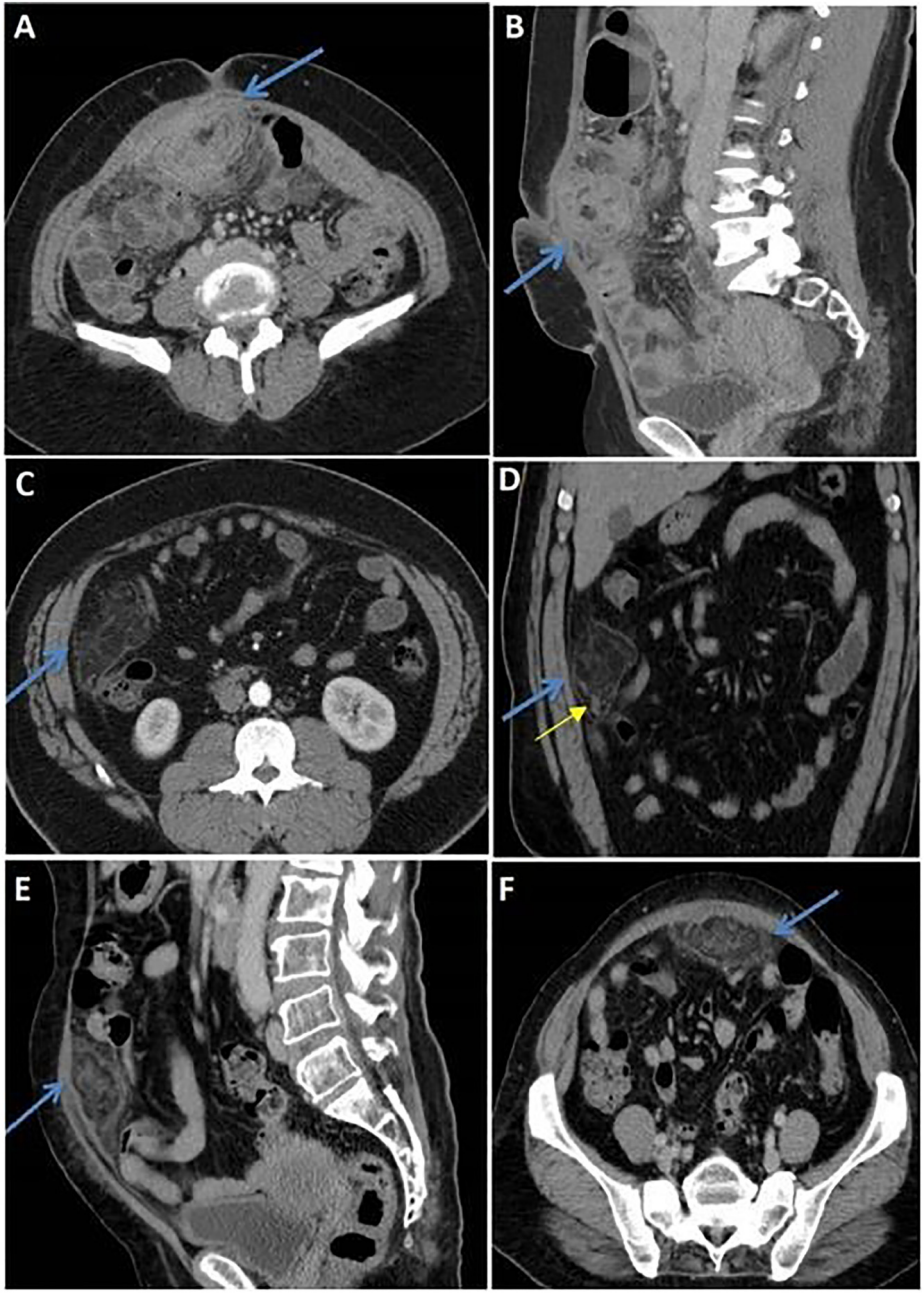
Radiologic findings. (A, B) CT coronal and sagittal view shows a focal soft tissue density area with surrounding fat stranding with omental vessels swirling within. (C, D) CT axial and coronal view showing extensive localised fat stranding lateral to the ascending colon and inferior to the right lobe of the liver with a thin peripheral hyperdenserim. Linear vertical collection of fluid attenuation is seen – omental infarct with a small abscess (yellow arrow). (E, F) CT sagittal and axial views showing an encapsulated omental infarct in the hypogastric region.

21 patients underwent a contrast CT scan of the abdomen, and one had an MRI scan. 80 % of patients had an ill-defined omental fat stranding on CT. The mean size of OI on CT was 6.04 cm (range: 4.0–8.0 cm). 4.7 % (one patient) had an abscess along with OI. The least common findings include oval-shaped, swirl-shaped, and encapsulated infarct OI changes on CT (Figure 2) (Table 2).

**Table 2:** Radiological characteristics.

Radiological variables	n (%)
Imaging modality	
CT	21 (95.45)
MRI	01 (4.5)
CT features	
Maximum size, cm	6.4 (SD ± 1.70)
Shape and complications	
Ill defined	17 (80)
Oval	1 (4.7)
Swirl	1 (4.7)
Encapsulated	1 (4.7)
Abscess	1 (4.7)

#### BMI and OI

The mean BMI observed was 26.56 kg/m^2^ (SD ± 3.21 kg/m^2^). 61.9 % belonged to class I, and 9.5 % had class II obesity per the Asia-Pacific body mass index classification. Mann-Whitney U tests were performed on the weight and BMI of the patients to correlate with the severity and size of the OI (greater than or less than 5 cm). Differences were considered insignificant with p-values>0.05 (Table 3).

**Table 3:** Obesity and Primary OI.

Obesity	Size of OI on CT	n^a^	Mean	SD	p-Value
BMI, kg/m^2^	<5 cm	11	26.99	3.98	0.53
	>5 cm	10	26.09	2.21	
Weight, kg	<5 cm	11	79.96	12.38	0.09
	>5 cm	10	71.33	9.92	

BMI, body mass index; n, number; SD, standard deviation. ^a^One patient’s body weight and BMI, was not retrievable for analysis.

### Management

Most patients had mild OI (81.8 %, n=18) and were treated successfully on an outpatient department (OPD) basis. Four patients (18.2 %) had moderate OI and required in-hospital admission due to pain not being relieved with oral analgesics and the need for parenteral treatment. One patient (4.5 %) had severe disease and required an emergency laparotomy and omentectomy, given the worsening clinical condition with features of shock and peritonism. Common OPD treatments included analgesics and anti-spasmodic medications. A course of either oral or parenteral antibiotics was prescribed in most patients (54.5 %). The resolution rate with conservative treatment was 95.45 % (n=21), and there were no recurrences on follow-up.

## Discussion

OI has a very low incidence of approximately 0.3 % of all patients presenting with acute abdomen to the emergency departments and is found in 0.1 % of laparotomies for acute abdominal pain [5]. Although commonly seen in pediatric age groups, it is also prevalent in the fifth decade in adults and seen twice as frequently in males than in females (male-to-female ratio of 2:1). Similar findings were observed in our study with a mean age of 47.45 years and 63.6 % being males. Abdominal pain is the cardinal presenting feature. Pain is often described as sudden onset, non-radiating, and frequently located on the right side of the abdomen (88 %) [6]. Our study also showed that pain was more common on the right side (50 %), including the right hypochondrium, right lumbar, and right iliac fossa. Other associated symptoms include nausea, vomiting, or fever. Acute intestinal obstruction, abscess formation, and peritonitis with or without bowel perforation are rare complications that should be ruled out in all cases of OI. OI was first categorized into primary and secondary by a landmark paper by Leitner et al. in 1951 [7]. The pathology of primary OI needs to be better understood. Numerous theories were proposed on alteration in omental vasculature resulting in venous stasis with hemorrhage and necrosis. A definitive pathology is often seen in secondary OI; these include internal hernias, adhesions, trauma, neoplasms, and systemic conditions predisposing to pro-thrombotic states. Anatomical variations in omental fat deposition play an essential role in OI. Obesity is a significant risk factor for developing OI [8].

A 10-year collective case series from four hospitals in Australia by Diab J et al. is one of the few sizeable available series on primary OI with 61 patients. 49.1 % of their patients were obese, the frequency of laparoscopic omentectomy was 31.1 % (n=19/61), and 8.1 % underwent a laparoscopic exploration without prior radiological imaging like CT [9]. The majority of our patients were obese, but a poor correlation existed between the patient’s weight or BMI and the severity of OI. These findings were similar to the analysis observed in the Diab J et al. series. Although literature reviews and case series mention obesity as a significant risk factor, omental fat or visceral fat percentage measurements on radiological imaging might correlate better with the severity of OI. Other risk factors are conditions leading to raised intra-abdominal pressure, local trauma, vascular congestion due to heart failure, and extreme exercise [8]. A literature review demonstrates that the right side of the omentum is affected in 80–90 % of cases [6, 10]. A long and easily mobile right omentum predisposes to torsion along the long axis and vascular compromise, leading to OI [11]. Grossly, the omentum appears congested, hemorrhagic, or sometimes necrotic and is wedge-shaped.

Lab investigations are often non-specific and may only indicate a state of acute inflammation with elevated WBC, ESR, and C-reactive protein levels. There is a poor correlation between the severity of OI and lab parameters. CT scan is considered the gold standard radiological investigation of choice for diagnosis [12]. It not only helps in confirming the diagnosis but excludes other acute conditions masqueraded by OI, like acute cholecystitis, appendicitis, strangulated internal hernias, or cysts. CT scan is also helpful in identifying secondary causes of OI. Secondary OI needs prompt recognition as they need definitive surgery.

**Figure 3:**
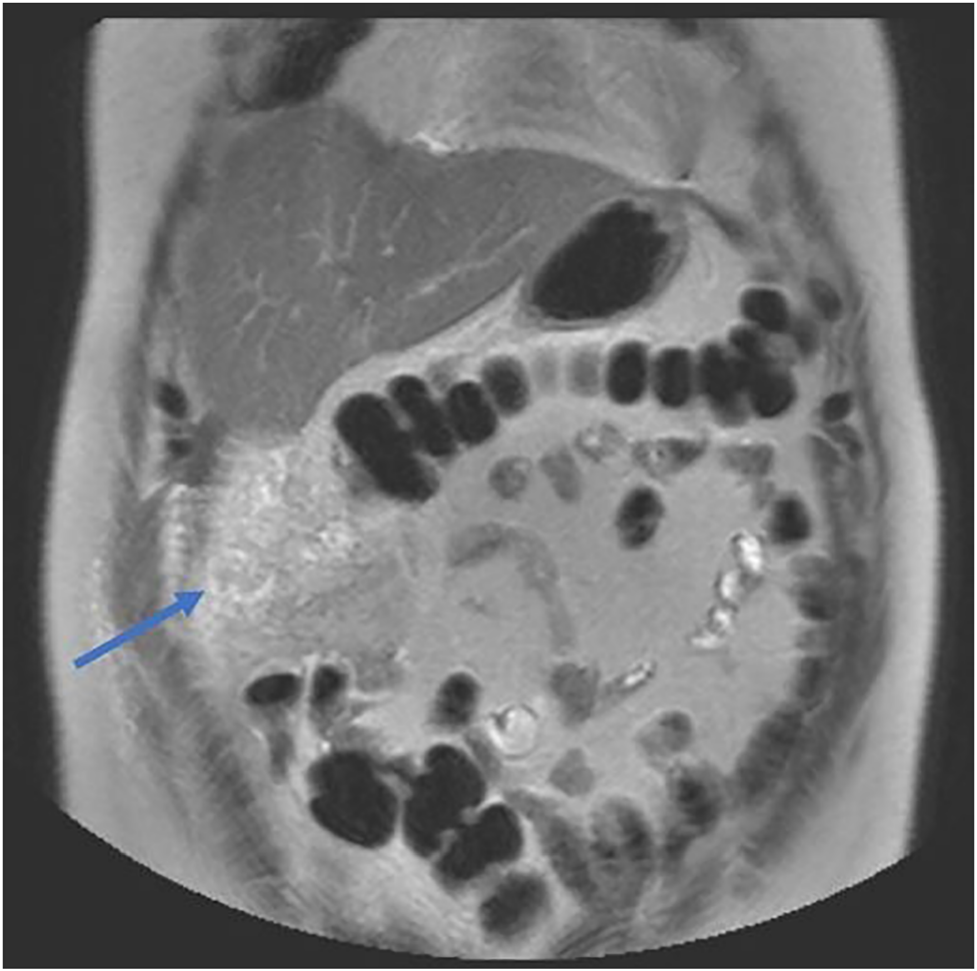
MRI T2 phase showing ill-defined roughly triangular area of altered signal intensity (blue arrow) appearing heterogeneously hyper intense in the omentum of right lumbar region just inferior to the right lobe of liver.

CT features are dynamic and change with the duration of the infarct. An ill-defined, heterogenous fat density lesion with no continuity is often seen in the early stage (<15 days) [13]. There was progression to well-defined, smaller lesions with a hyperdense rim when imaging was done beyond two weeks [13]. Our study’s mean duration of symptoms was 8.64 days, and a large majority had radiological imaging in the above-described early period. Similar to earlier studies and radiological case reports, ill-defined omental fat stranding localized to a specific abdominal quadrant was seen on CT imaging in 81.8 % of patients in our study, and one patient progressed to develop an abscess inside the OI. Varied shapes of OI were also observed in our study: oval-shaped and swirl-shaped, along with omental vessels and encapsulated OI. MRI imaging classically shows altered signal intensity in T2 phases, as seen in one patient from our study (Figure 3).

Imaging differentials are diverticulitis and epiploic appendagitis. Omental infarct and epiploic appendagitis are a spectrum of conditions grouped under Intraperitoneal Focal Fat Infarction, as pathophysiology remains the same for both. However, on imaging, epiploic appendages are usually smaller, and the standard location is the rectosigmoid junction. Conversely, the omental infarct is most common along the ascending colon and is more significant than 3 cm in size. Central fat density having a hyperdense rim with a central dot is the classical appearance of epiploic appendagitis.

Management of OI is a topic for debate. There is a paradigm shift from the earlier surgical approach of performing a laparotomy or laparoscopy to a more conservative management in OI [14]. According to an extensive systematic review by Medina-Gallardo NA et al., successful resolution of OI was seen in 84.1 % with a conservative approach using analgesics [4]. Our study showed that 95.5 % managed conservatively without treatment failure or recurrence. The surgical intervention rate in our study was 4.5 %, which is much lower compared to the Diab J et al. series (31.1 %) and Medina-Gallardo NA et al. review (25.7 %) [4, 9]. About 50 % of our study population were treated with an oral course of antibiotics and analgesics to prevent progression to sepsis or local abscess formation. However, more randomized controlled trials are needed to justify its usage.

## Conclusions

OI, even though rare, should be kept in mind in patients presenting with acute or subacute pain in the abdomen. CT or MRI scan is essential in diagnosis and excluding other causes. The majority runs a self-limiting course, so conservative management should be OI’s first management line.
